# OVOL1/2 regulate trophectoderm differentiation during the morula-to-blastocyst transition

**DOI:** 10.1038/s42003-026-11168-x

**Published:** 2026-10-29

**Authors:** Yi Zhu, Xueqin Chen, Xin Deng, Shuoping Zhang, Jue Zhang, Fei Meng, Qinwei Zhou, Yifan Gu, Fei Gong, Guangxiu Lu, Ge Lin, Lizhi Leng

**Affiliations:** 1 NHC Key Laboratory of Human Stem Cell and Reproductive Engineering, Xiangya School of Basic Medical Sciences, Central South University, Changsha, China; 2 Clinical Research Center for Reproduction and Genetics in Hunan Province, Reproductive and Genetic Hospital of CITIC-XIANGYA, Changsha, China; 3 National Engineering and Research Center of Human Stem Cell, Changsha, China

**Keywords:** Embryology, Epithelial-mesenchymal transition

## Abstract

The blastocyst formation rate of human embryos cultured in vitro for assisted reproduction is approximately 50%, with most embryos arresting at the cleavage stage; however, the underlying mechanisms remain poorly understood. Here, we performed single-cell RNA sequencing of biopsied blastomeres from morphologically high-quality 8-cell embryos and tracked the developmental progression of the remaining embryos to the blastocyst stage. Embryos that failed to form blastocysts exhibited impaired zygotic genome activation (ZGA). We identified *OVOL1/2* as critical ZGA genes that were markedly underactivated in these embryos. Functional analyses in both mouse and human embryos demonstrate that depletion of OVOL1/2 disrupts trophectoderm differentiation via ARPC1B-dependent mechanisms, thereby impairing the morula-to-blastocyst transition. Collectively, these findings provide mechanistic insights into preimplantation embryonic development and reveal potential causes of blastocyst formation failure.

## Abstract (web summary)

Blastocyst formation failure is a major challenge in preimplantation development. We identify OVOL1/2 as regulators of the morula-to-blastocyst transition that promote trophectoderm differentiation through ARPC1B-mediated cytoskeletal remodeling.

## Introduction

During early mammalian embryonic development, the initial cleavage divisions depend predominantly on maternally derived transcripts, such as MATER^1^, TLE6^2^, BTG4^3^, CNOT6L^4^, and ZAR1/2^5^. Abnormalities in these maternal factors are commonly associated with embryonic arrest at the 2- to 8-cell stages. As maternal transcripts are progressively degraded, zygotic genome activation (ZGA) occurs, enabling the embryo to undergo compaction and polarization and to form a tightly organized morula, which subsequently develops into a blastocyst comprising the inner cell mass (ICM) and trophectoderm (TE). Thus, ZGA is essential for embryo progression beyond the cleavage stages. Transcriptome sequencing studies of early embryos have revealed that thousands of genes are activated during ZGA. Among these, genes such as *ZSCAN4*^6^, *DUX4*^7^, *TPRX1/2*^8^, and *MERVL*^9^ play pivotal roles in early embryonic development. *ZSCAN4*^6^ protects DNA from damage and maintains genomic stability; *DUX4*^7^ and *TPRX1/2*^8^ regulate chromatin accessibility; and *MERVL*^9^ participates in chromatin remodeling and the transition toward pluripotency. Aberrant expression of these genes has been linked to impaired blastocyst formation. However, the molecular mechanisms through which ZGA genes regulate early embryonic development remain incompletely understood.

Compaction and polarization are critical morphological events during preimplantation embryonic development, occurring between the 8-cell and morula stages. The establishment of apical-basal polarity enables blastomeres to generate daughter cells that either maintain or lose polarity during subsequent asymmetric divisions^10^. Meanwhile, differences in adhesion and cortical tension between adjacent cells promote cell internalization, leading to the formation of distinct populations of polarized outer cells and apolar inner cells^11^. During this process, Hippo signaling and lineage-specific gene expression, regulated by cell position^12^ and polarity^13^, contribute to the segregation of the ICM and TE lineages^14^.

OVOL proteins are evolutionarily conserved C2H2 zinc-finger transcription factors (TFs) found from metazoans to vertebrates. Vertebrates possess three Ovol genes, *Ovol1–3*, among which *Ovol1* and *Ovol2* are the most extensively studied. Previous studies have demonstrated that OVOL1/2 promote the mesenchymal-to-epithelial transition (MET)^15–17^. Specifically, OVOL1/2 cooperate to induce actin redistribution in epithelial cells, initiate cytoskeletal remodeling, and facilitate MET by promoting the establishment of intercellular junctions^18^. Notably, compaction and polarization in preimplantation embryos resemble MET-like processes, during which blastomeres progressively establish apical–basal polarity through the activation of apical polarity proteins^19,20^. Transcriptomic analyses have shown that *OVOL1/2* are upregulated during ZGA in both mouse and human preimplantation embryos^21,22^. However, whether OVOL1/2 similarly promote MET-like processes in early embryos and thereby contribute to blastocyst formation remains unclear.

In this study, we demonstrate that the failure of morphologically high-quality 8-cell embryos to develop into blastocysts is associated with impaired ZGA. *OVOL1/*2 were identified as key ZGA genes, and their insufficient activation contributes to failure of the morula-to-blastocyst transition. These findings expand our understanding of the molecular mechanisms governing preimplantation embryonic development and provide mechanistic insight into why a subset of morphologically high-quality cleavage-stage embryos fail to progress to the blastocyst stage.

## Results

### Morphologically high-quality 8-cell embryos that fail to form blastocysts exhibit impaired zygotic genome activation

**Fig. 1:**
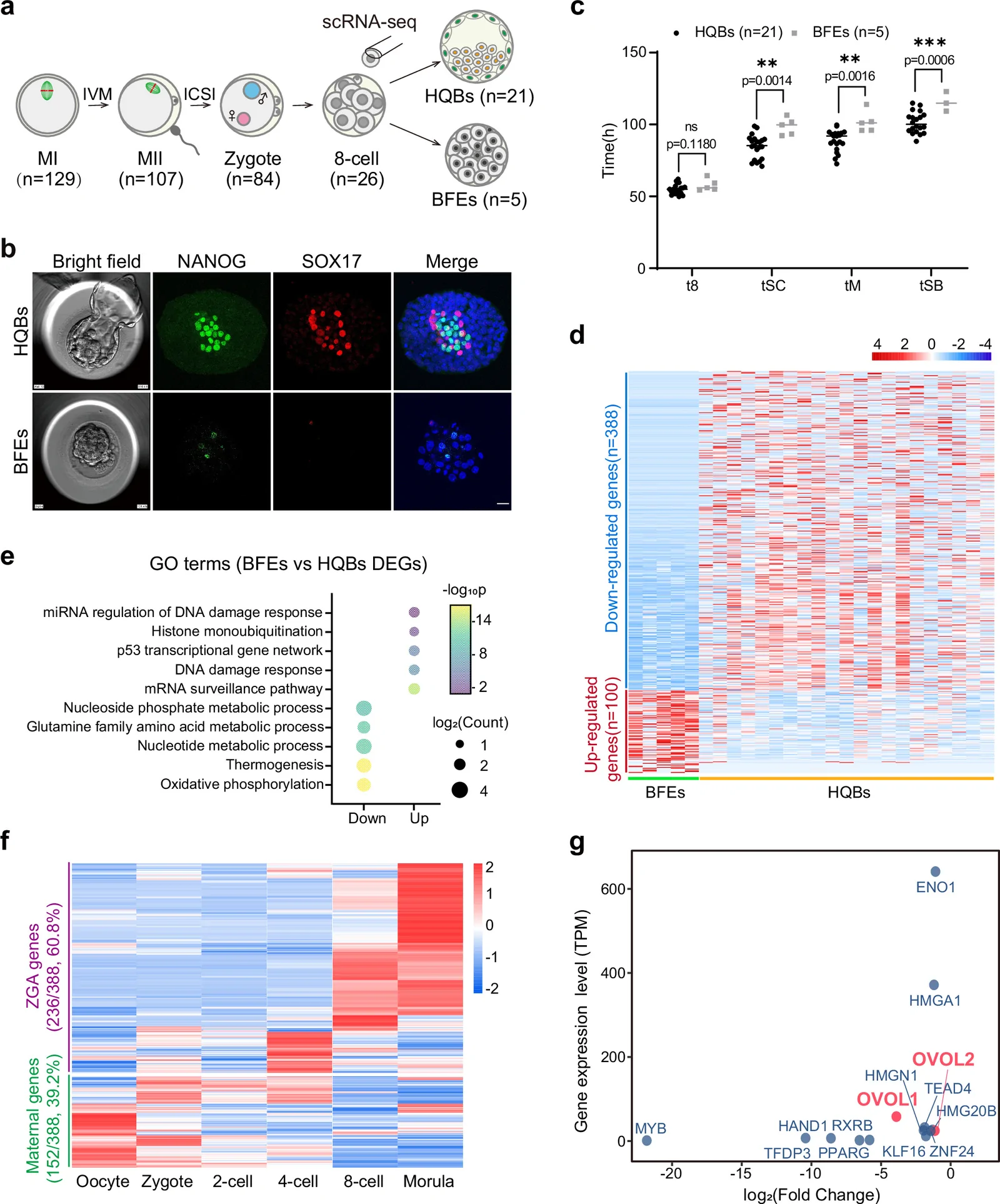
Morphologically high-quality 8-cell embryos that fail to form blastocysts exhibit impaired zygotic genome activation. **a** Schematic of the experimental workflow. *n* indicates the number of samples analyzed per stage and per group. **b** Confocal immunofluorescence images of high-quality blastocysts (HQBs) and blastocyst formation failure embryos (BFEs) at the blastocyst stage, showing NANOG (green) and SOX17 (red). DNA was counterstained with DAPI (blue). Scale bar, 25 μm. **c** Morphokinetic timeline of embryonic development, indicating the timing of the 8-cell stage (t8), onset of compaction (tSC), morula formation (tM), and blastocyst formation (tSB) in HQBs and BFEs. All values are presented in hours (h). Parameters include tn (time to reach the n-cell stage); tSC (onset of compaction); tM (completion of compaction); and tSB (onset of blastocyst formation). Data are presented as mean ± SD. The number of embryos analyzed is indicated (n). n.s. not significant, **P < 0.01, ***P < 0.001, by two-tailed Student’s *t*-test. **d** Heatmap displaying differentially expressed genes (DEGs) between HQBs and BFEs. **e** Bubble plot illustrating Gene Ontology (GO) enrichment analysis of DEGs identified in BFEs. **f** Expression dynamics of DEGs downregulated in BFEs across human preimplantation developmental stages. **g** Scatter plot depicting differential gene expression between HQBs and BFEs, with 14 transcription factors downregulated in BFEs highlighted.

During in vitro fertilization (IVF), some patients generate multiple morphologically high-quality cleavage-stage embryos on day 3 (D3), yet experience developmental failure following D3 embryo transfer and/or extended blastocyst culture. To investigate the mechanisms underlying blastocyst formation failure in these embryos, immature metaphase I (MI) oocytes were collected, matured in vitro to the metaphase II (MII) stage, and subsequently fertilized. The resulting zygotes were monitored using a time-lapse imaging system. A single blastomere was biopsied from each 8-cell embryo that exhibited no obvious abnormalities during the first three cleavage divisions and was subjected to single-cell RNA sequencing (scRNA-seq). The corresponding source embryos were then cultured further to the blastocyst stage. In total, 129 MI oocytes were collected, yielding 107 MII oocytes, 84 zygotes, and 26 biopsied 8-cell embryos. Among these, 21 embryos successfully developed into high-quality blastocysts (HQBs), defined as blastocysts with an ICM and/or TE graded A or B according to the Gardner scoring system^23^. The remaining five embryos either failed to form blastocysts or developed into blastocysts in which both the ICM and TE were graded C; these embryos were therefore classified as blastocyst formation failure embryos (BFEs) (Fig. 1a, b). Compared with HQBs, BFEs exhibited a progressively slower developmental pace at three key stages: initiation of compaction (tSC), morula formation (tM), and blastocyst initiation (tSB) (*P* < 0.01; Fig. 1c and Supplementary Table 1).

We next analyzed differentially expressed genes (DEGs) between HQBs and BFEs. Compared with HQBs, BFEs showed 100 upregulated and 388 downregulated genes (Fig. 1d). Gene Ontology (GO) enrichment analysis revealed that the upregulated DEGs in BFEs were predominantly associated with mRNA surveillance pathways and DNA damage responses, whereas the downregulated DEGs were enriched in pathways related to oxidative phosphorylation and amino acid metabolism (Fig. 1e). Integration of these DEGs with published human embryo scRNA-seq datasets^22^ demonstrated that more than 60% of the downregulated DEGs normally exhibit progressive upregulation from the 4-cell to morula stage (Fig. 1f). These findings suggest that these genes follow a ZGA-associated expression trajectory during human embryonic development, and that their reduced expression in BFEs reflects insufficient ZGA. Given the central role of TFs in orchestrating gene expression programs, we examined TFs among the downregulated DEGs and identified 13 candidates, including HMGA1^24^, PPARG^25^, HAND1^26^, and TEAD4^27^, which have been implicated in oxidative phosphorylation and trophoblast differentiation. In addition, KLF16^28^, ZNF24^29^, and MYB^30^ were associated with cell proliferation and differentiation. Notably, *OVOL1/2* were markedly downregulated in BFEs, suggesting that they may represent novel ZGA genes that are indispensable for blastocyst formation (Fig. 1g).

### OVOL1/2 are essential for mouse blastocyst formation

Transcriptomic analyses of mouse and human preimplantation embryos revealed that OVOL1 and its homolog OVOL2 are progressively upregulated during the 2- to 4-cell stages in mice and the 4- to 8-cell stages in humans^21,22^ (Supplementary Fig. 1a, b). Immunofluorescence staining revealed that OVOL1 and OVOL2 are first detectable at the 2-cell stage in mouse embryos, with expression levels progressively increasing thereafter. At the 2- to 4-cell stages, both proteins localize to both the cytoplasm and nucleus, but gradually shift toward predominantly nuclear localization as development proceeds, becoming largely nuclear by the blastocyst stage. In human embryos, OVOL1 remains localized to both the cytoplasm and nucleus throughout preimplantation development. In contrast, OVOL2 is primarily localized in the cytoplasm at the 8-cell and morula stages, but becomes predominantly nuclear by the blastocyst stage (Supplementary Fig. 1c).

**Fig. 2:**
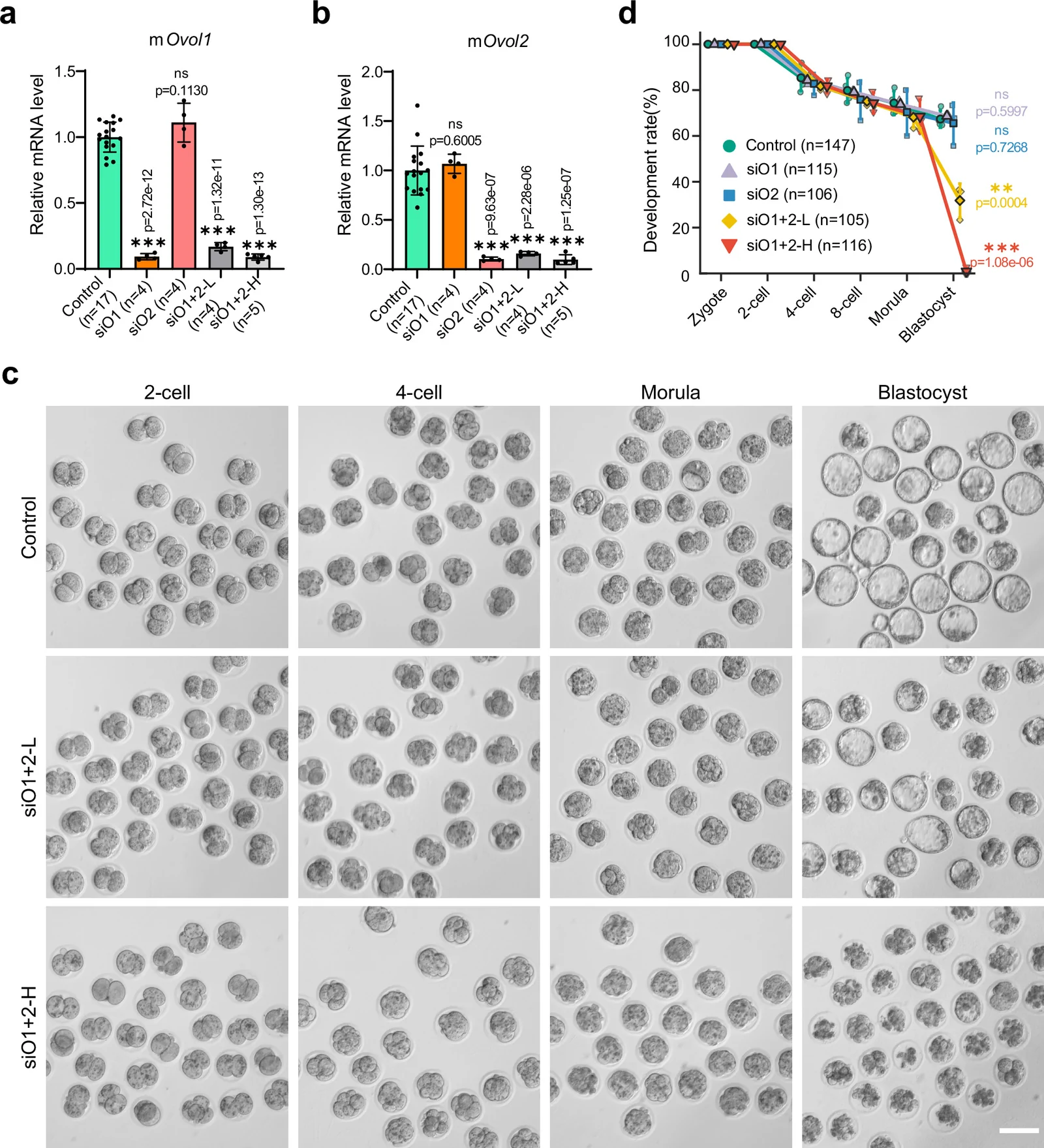
OVOL1/2 are essential for mouse blastocyst formation. **a**,**b** RT‑PCR quantification of *Ovol1* (**a**) and *Ovol2* (**b**) mRNA levels in 2‑cell embryos after siRNA microinjection. *β‑actin* served as an internal control. Data are presented as mean ± SD, normalized to the control group (set to 1.0). For each sample, two embryos were pooled, and the number of biological replicates analyzed is indicated (n). **c** Differential interference contrast (DIC) images of embryos at the 2‑cell, 4‑cell, morula, and blastocyst stages following siRNA treatment. Scale bar, 100 μm. n.s. not significant, ***P < 0.001, by two-tailed Student’s *t*-test. **d** Developmental progression of embryos after hCG administration. The number of embryos analyzed is indicated (n). Data are presented as mean ± SD. n.s. not significant, **P < 0.01, ***P < 0.001, by two-tailed Student’s *t*-test.

To investigate the functions of OVOL1 and OVOL2 during preimplantation development, we performed siRNA-mediated knockdown at the zygote stage in mouse embryos. Quantitative RT–PCR analysis confirmed robust reductions in *Ovol1* and *Ovol2* transcript levels in the si*Ovol1* (siO1) and si*Ovol2* (siO2) groups, respectively, at the 2-cell stage (Fig. 2a, b). After 3.5 days of in vitro culture, no significant differences in developmental rates were observed among the control, siO1, and siO2 groups (Fig. 2d and Supplementary Fig. 2a). We next co-injected siRNAs targeting both *Ovol1* and *Ovol2* to generate low-dose (siO1 + 2-L, 120 μM) and high-dose (siO1 + 2-H, 200 μM) double-knockdown groups. Both conditions led to pronounced suppression of OVOL1 and OVOL2 at the mRNA and protein levels (Fig. 2a, b and Supplementary Fig. 2b, c). Embryos in both the siO1 + 2-L and siO1 + 2-H groups developed normally to the morula stage. However, the blastocyst formation rate was significantly reduced in siO1 + 2-L embryos, whereas siO1 + 2-H embryos failed to progress beyond the morula stage (Fig. 2c, d). Collectively, these findings suggest partial functional redundancy between OVOL1 and OVOL2 and demonstrate that OVOL1/2 activity is indispensable for the morula-to-blastocyst transition.

### OVOL1/2 knockdown impairs trophectoderm differentiation

**Fig. 3:**
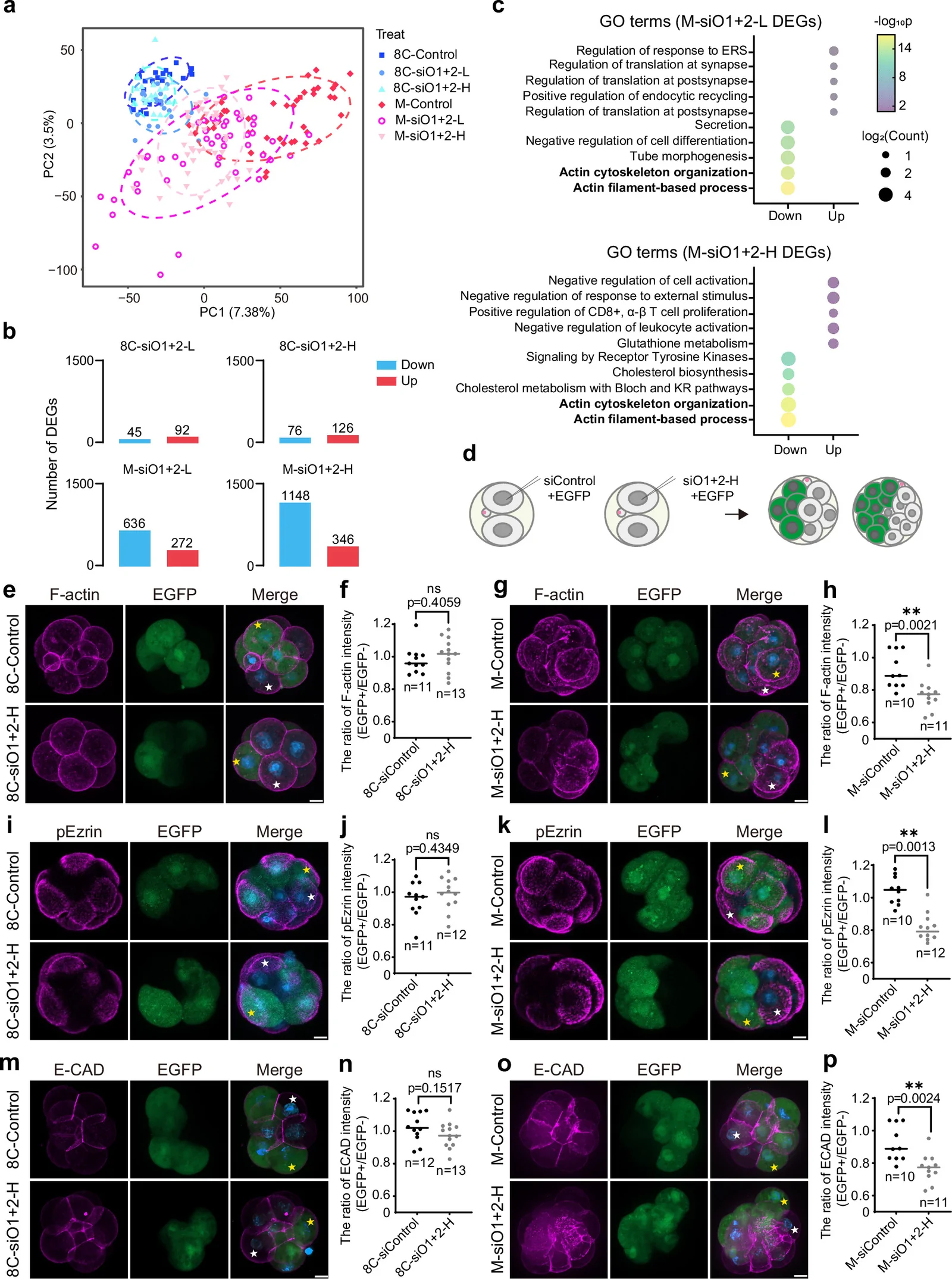
Knockdown of OVOL1/2 disrupts polarization and compaction in early mouse embryos. **a** Principal component analysis (PCA) of siO1 + 2 8-cell and morulae. **b** Histogram showing the number of DEGs in siO1 + 2 8-cell and morulae (red, upregulated; blue, downregulated). **c** GO enrichment analysis of DEGs in siO1 + 2 morulae. **d** Schematic of 2-cell microinjection. **e**,**g**,**i**,**k**,**m**,**o** Single blastomeres from mouse 2‑cell embryos were co‑injected with EGFP mRNA and either siControl or siO1 + 2‑H siRNA, then cultured to the 8‑cell and morula stages for immunofluorescence staining. Confocal images showing F‑actin (magenta) (**e**,**g**), pEzrin (magenta) (**i**,**k**), and E‑CAD (magenta) (**m**,**o**) in control and siO1 + 2 8‑cell and morula embryos. DNA was counterstained with DAPI (blue). Scale bar, 25 μm. Yellow and white stars indicate EGFP‑positive and EGFP‑negative blastomeres, respectively. **f**,**h**,**j**,**l**,**n**,**p** Quantification of fluorescence signal intensity for F‑actin (**f**,**h**), pEzrin (**j**,**l**), and E-CAD (**n**,**p**) in 8‑cell and morula embryos. The number of embryos analyzed is indicated (n). Data are presented as mean ± SD. n.s. not significant, ***P < 0.001, by two-tailed Student’s *t*-test.

To elucidate the molecular mechanisms underlying blastocyst formation failure in *Ovol1/2*-knockdown embryos, we collected 8-cell and morula-stage embryos for scRNA-seq analysis. Principal component analysis (PCA) identified two distinct subclusters corresponding to developmental stage, with greater transcriptomic heterogeneity at the morula stage than at the 8-cell stage (Fig. 3a). Consistent with this observation, the number of DEGs between siO1 + 2-L/H and control embryos was markedly higher at the morula stage than at the 8-cell stage. Notably, among siO1 + 2-L/H morulae, a substantially larger proportion of DEGs were downregulated rather than upregulated relative to controls (Fig. 3b). GO enrichment analysis showed that the downregulated DEGs in siO1 + 2-L/H morulae were significantly enriched in pathways related to actin cytoskeleton organization and actin filament-based processes (Fig. 3c). Consistently, transcriptomic profiling revealed a significant reduction in the expression of actin-related genes in siO1 + 2-L/H morulae (Supplementary Fig. 3a). To further investigate the effects of OVOL1/2 knockdown on F-actin expression and localization, we co-injected siO1 + 2-H and EGFP mRNA into a single blastomere of mouse 2-cell embryos (Fig. 3d). Control embryos received co-injection of siControl and EGFP mRNA into one blastomere. Immunofluorescence staining showed that F-actin was localized beneath the blastomere membrane in both control and siO1 + 2-H embryos. At the 8-cell stage, F-actin fluorescence intensity did not differ significantly between EGFP-positive and EGFP-negative blastomeres in either group. Similarly, in control morulae, F-actin fluorescence intensity remained comparable between EGFP-positive and EGFP-negative blastomeres. In contrast, in siO1 + 2-H morulae, F-actin fluorescence intensity was significantly reduced in EGFP-positive blastomeres relative to EGFP-negative blastomeres, confirming that OVOL1/2 knockdown impairs F-actin expression (Fig. 3e–h).

During early embryonic development, cytoskeletal components such as actin and keratin are thought to sense internal and external signals that promote the formation and maintenance of the apical domain^31,32^. Transcriptomic analysis further revealed significant downregulation of genes associated with polarization and compaction in siO1 + 2-L/H morulae. Western blot analysis confirmed marked reductions in pEzrin and E-cadherin protein levels in siO1 + 2-H morulae. By contrast, no significant differences in the expression of polarization- or compaction-related genes or proteins were observed between *Ovol1/2*-knockdown and control embryos at the 8-cell stage (Supplementary Fig. 3b–e). To assess the impact of OVOL1/2 knockdown on the establishment of embryonic polarity and compaction, we again co-injected siO1 + 2-H and EGFP mRNA into a single blastomere of mouse 2-cell embryos. Immunofluorescence analysis showed that pEzrin localized to the apical domain, whereas E-CAD was enriched at the basolateral membrane. At the 8-cell stage, neither the expression levels nor the subcellular localization of pEzrin and E-CAD differed significantly between EGFP-positive and EGFP-negative blastomeres in either control or siO1 + 2-H embryos. Similarly, in control morulae, pEzrin and E-CAD expression and localization were comparable between EGFP-positive and EGFP-negative blastomeres. In contrast, in siO1 + 2-H morulae, the fluorescence intensities of both pEzrin and E-CAD were markedly reduced in EGFP-positive blastomeres compared with EGFP-negative blastomeres (Fig. 3i–p). Collectively, these findings indicate that OVOL1/2 knockdown disrupts the establishment of embryonic polarity and impairs compaction.

**Fig. 4:**
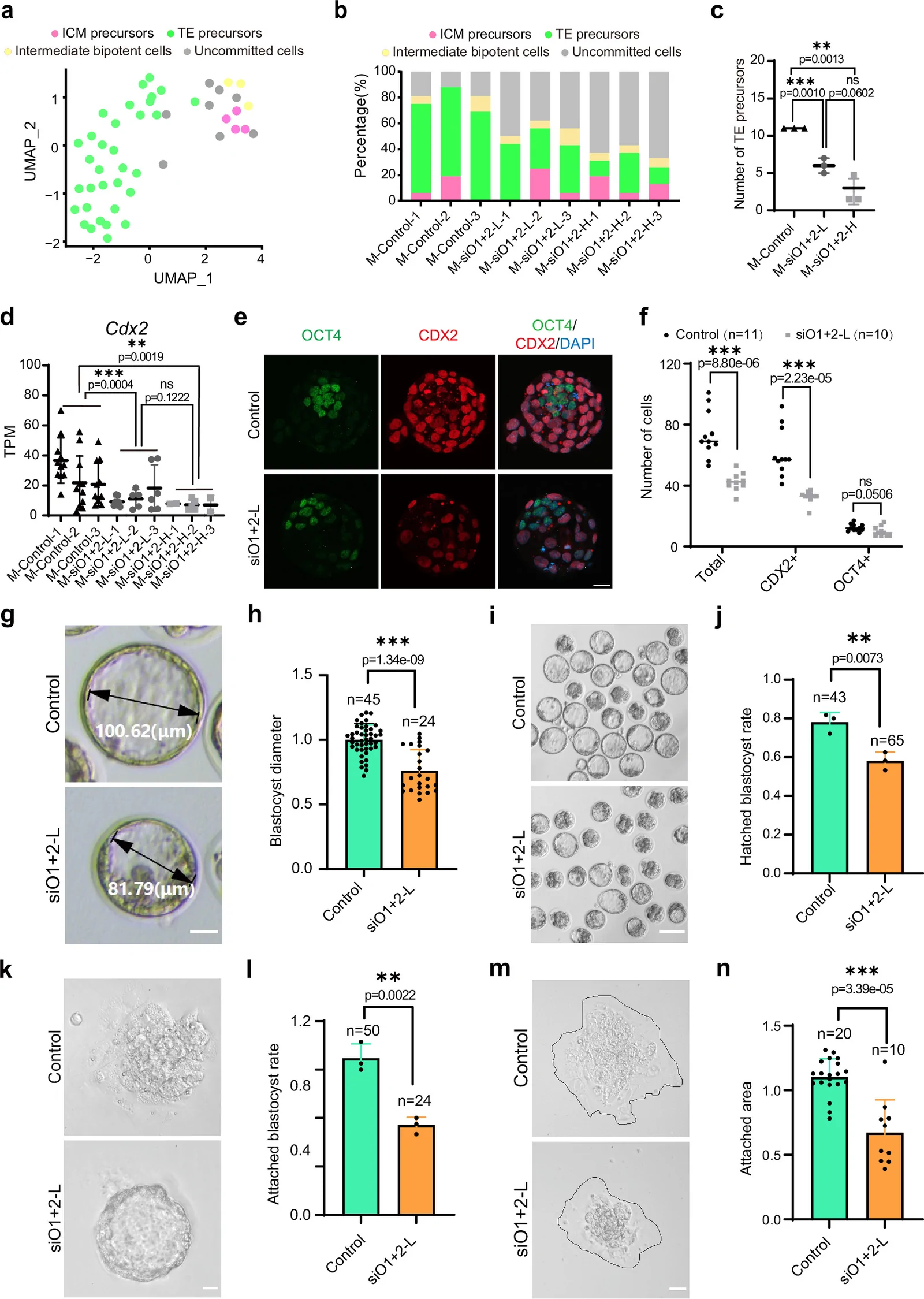
Impaired trophectoderm differentiation in OVOL1/2 knockdown embryos. **a** UMAP clustering of control morulae based on *Sox2* and *Cdx2* expression. Pink dots: S*ox2* positive cells (ICM precursors), green dots: *Cdx2* positive cells (TE precursors), yellow dots: *Sox2*/*Cdx2* double-positive cells (intermediate bipotent cells), and gray dots: *Sox2*/*Cdx2* double-negative cells (uncommitted cells). **b** Proportions of ICM precursors, TE precursors, intermediate bipotent cells, and uncommitted cells in control and siO1 + 2 morulae. **c**,**d** scRNA-seq quantification of TE precursor proportions (**c**) and *Cdx2* expression levels (**d**) in control and siO1 + 2 morulae. n.s. not significant, **P < 0.01, ***P < 0.001, by two-tailed Student’s *t*-test. **e** Confocal images of CDX2 (red) and OCT4 (green) in control and siO1 + 2-L blastocysts. DNA was counterstained with DAPI (blue). Scale bar, 25 μm. **f** Quantification of CDX2-positive and OCT4-positive cells. The number of embryos analyzed is indicated (n). Data are presented as mean ± SD. n.s. not significant, ***P < 0.001, by two-tailed Student’s *t*-test. **g** Representative images illustrating lumen diameter measurements. Scale bar, 25 μm. **h** Quantification of lumen diameter. The number of embryos analyzed is indicated (n). Data are presented as mean ± SD, normalized to the control group (set to 1.0). ***P < 0.001, by two-tailed Student’s *t*-test. **i** DIC images of blastocysts following the indicated siRNA treatments. Scale bar, 100 μm. **j** Percentage of hatched blastocysts. The number of embryos analyzed is indicated (n). Data are presented as mean ± SD. **P < 0.01, by two-tailed Student’s *t*-test. **k** DIC images of control and siO1 + 2-L blastocysts attached to culture plates after 24 h. Scale bar, 25 μm. **l** Percentage of attached blastocysts. The number of embryos analyzed is indicated (n). Data are presented as mean ± SD. **P < 0.01, by two-tailed Student’s *t*-test. **m** Representative images illustrating attachment area measurements. Scale bar, 25 μm. **n** Quantification of attachment area. The number of embryos analyzed is indicated (n). Data are presented as mean ± SD, normalized to the control (set to 1.0). ***P < 0.001, by two-tailed Student’s *t*-test.

Polarization drives the asymmetric distribution of cellular components and provides the foundation for ICM/TE lineage segregation during preimplantation development^10^. To determine the impact of *Ovol1/2* downregulation on lineage specification, we analyzed scRNA-seq data from control morulae and identified four distinct cell populations: *Sox2*-positive, *Cdx2*-positive, *Cdx2*/*Sox2* double-positive, and *Cdx2*/*Sox2* double-negative cells. These populations correspond to ICM precursor cells, TE precursor cells, intermediate bipotent cells, and uncommitted cells, respectively (Fig. 4a). The numbers of *Cdx2*-positive cells in control, siO1 + 2-L, and siO1 + 2-H morulae were 11, 6, and 3, respectively, corresponding to 68.8%, 37.5%, and 18.8% of total embryonic cells (Fig. 4b, c). These results indicate a marked reduction of TE precursor cells in siO1 + 2-L/H morulae compared with controls. Furthermore, *Cdx2* expression within TE precursor cells was significantly reduced in siO1 + 2-L/H morulae (Fig. 4d). To further evaluate the effects of OVOL1/2 knockdown on TE formation at the blastocyst stage, we performed immunofluorescence staining for CDX2 and OCT4 in siO1 + 2-L blastocysts. Compared with controls, siO1 + 2-L blastocysts exhibited a significant reduction in the number of CDX2-positive cells, whereas the number of OCT4-positive cells remained comparable (Fig. 4e, f). Consistently, siO1 + 2-L blastocysts displayed significantly smaller diameters (Fig. 4g, h). In outgrowth assays, 78.0% control blastocysts hatched from the zona pellucida within 24 h and 64.6% hatched control blastocysts attached to the culture surface by 48 h. In contrast, only 58.1% of siO1 + 2-L blastocysts hatched, and 63.1% of those that hatched failed to attach (Fig. 4i–l). Moreover, the blastocysts that successfully attached exhibited significantly smaller attachment areas compared with controls (Fig. 4m, n). Collectively, these data demonstrate that OVOL1/2 knockdown reduces TE cell number and compromises TE function.

### OVOL1/2 regulate embryonic development through ARPC1B

**Fig. 5:**
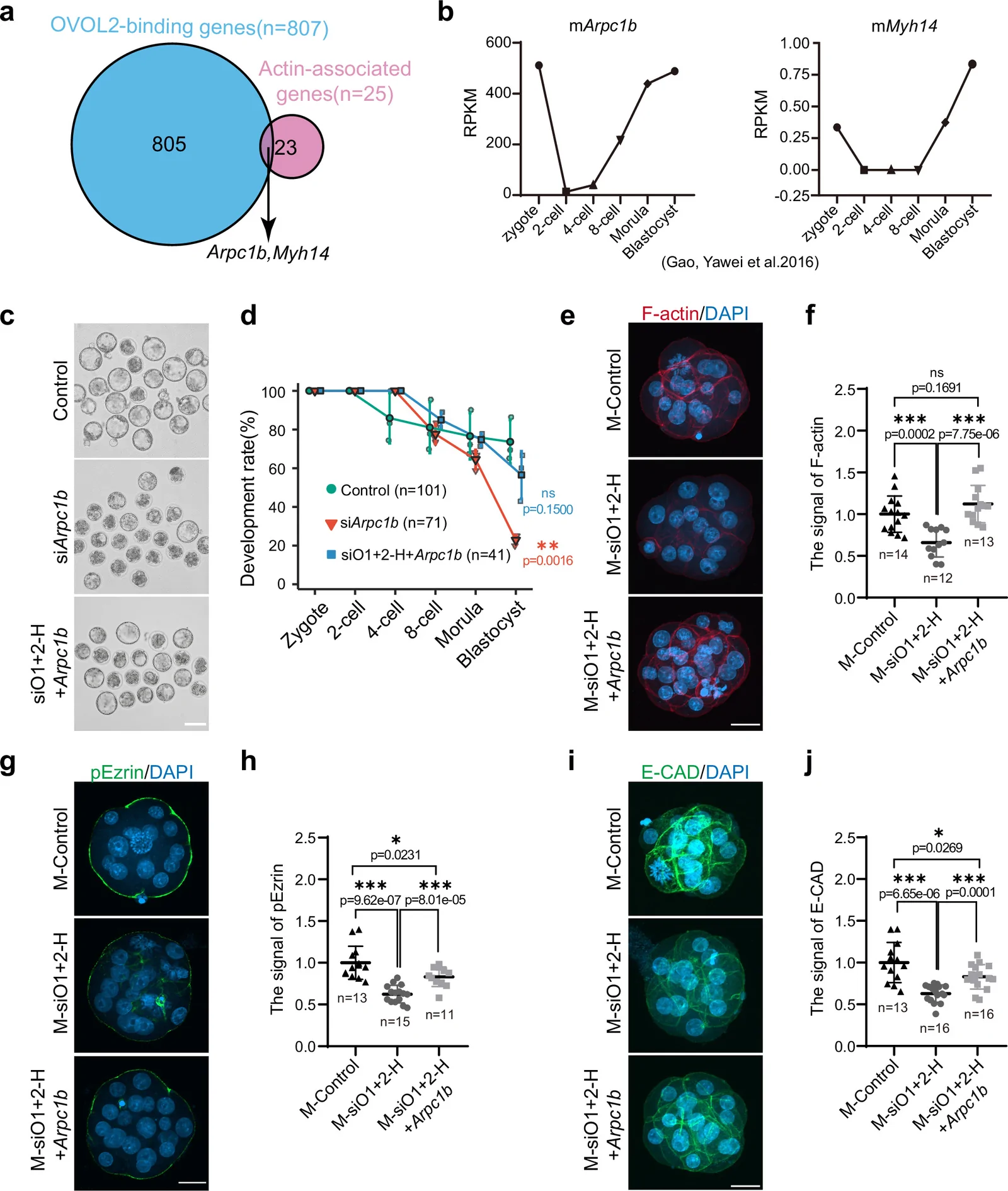
OVOL1/2 regulate trophectoderm differentiation through ARPC1B. **a** Venn diagram showing the overlap between OVOL2-bound genes and actin-related genes downregulated in siO1 + 2 morulae. **b** RNA-seq–derived expression of *Arpc1b* and *Myh14* in mouse embryos. **c** Representative DIC images of E3.5 embryos following siRNA microinjection or siRNA+mRNA electroporation. The number of embryos analyzed is indicated (n). Scale bar, 100 μm. **d** Developmental rates corresponding to (**c**). The number of embryos analyzed is indicated (n). Data are presented as mean ± S.D. n.s. not significant, **P < 0.01, by two-tailed Student’s *t*-test. **e**,**g**,**i** Representative confocal images of F-actin (**e**), pEzrin (**g**), and E-CAD (**i**) in control, siO1 + 2-H, and siO1 + 2-H+*Arpc1b* morulae. DNA was counterstained with DAPI (blue). Scale bar, 25 μm. **f**,**h**,**j** Quantification of F-actin (**f**), pEzrin (**h**), and E-CAD (**j**) signal intensity. The number of embryos analyzed is indicated (n). Data are presented as mean ± SD and normalized to control values. n.s. not significant, *P < 0.05, ***P < 0.001, by two-tailed Student’s *t*-test.

To elucidate the mechanisms by which OVOL1/2 regulate embryonic development, we integrated published OVOL2 ChIP-seq data^33^ with actin-associated genes that were downregulated in siO1 + 2-L/H embryos. This analysis identified two candidate targets, *Arpc1b* and *Myh14* (Fig. 5a). Given the minimal expression of *Myh14* during mouse preimplantation development, *Arpc1b* was considered the most plausible OVOL1/2 target (Fig. 5b). The ARP2/3 complex is a key regulator of actin cytoskeletal dynamics, mediating the nucleation of branched actin filaments and the formation of complex actin networks^34^. ARPC1B, a regulatory subunit of this complex, is essential for organizing nucleation sites and facilitating interactions with nucleation-promoting factors^35^. Immunofluorescence analysis revealed that ARPC1B is highly expressed in zygotes, decreases at the 2- to 4-cell stages, and increases again from the 8-cell stage to the blastocyst stage, exhibiting predominantly cytoplasmic localization in blastomeres (Supplementary Fig. 4a). These findings indicate that ARPC1B is derived from both maternal and embryonic sources. As *OVOL1/2* are ZGA genes, they are likely to regulate embryo-derived ARPC1B expression. To test this hypothesis, we electroporated si*Arpc1b* into 4-cell embryos to achieve knockdown of *Arpc1b* expression. The blastocyst formation rate in si*Arpc1b* embryos was markedly reduced to 22.8%, compared with 73.6% in controls (Fig. 5c, d), closely phenocopying the defect observed in siO1 + 2-L embryos. Consistently, Arpc1b expression was significantly reduced in siO1 + 2-L/H embryos (Supplementary Fig. 4b, c), supporting its role as a downstream effector of OVOL1/2. To further validate this regulatory relationship, we electroporated *Arpc1b* mRNA into siO1 + 2-H embryos at the 4-cell stage. *Arpc1b* supplementation partially rescued blastocyst development, increasing the formation rate to 56.5%, compared with 73.6% in controls (Fig. 5c, d). Moreover, F-actin organization was largely restored, and p-Ezrin and E-cadherin expression were partially recovered in siO1 + 2-H + *Arpc1b* morulae (Fig. 5e–j). Collectively, these results demonstrate that OVOL1/2 promote early embryonic development, at least in part, by upregulating ARPC1B expression.

**Fig. 6:**
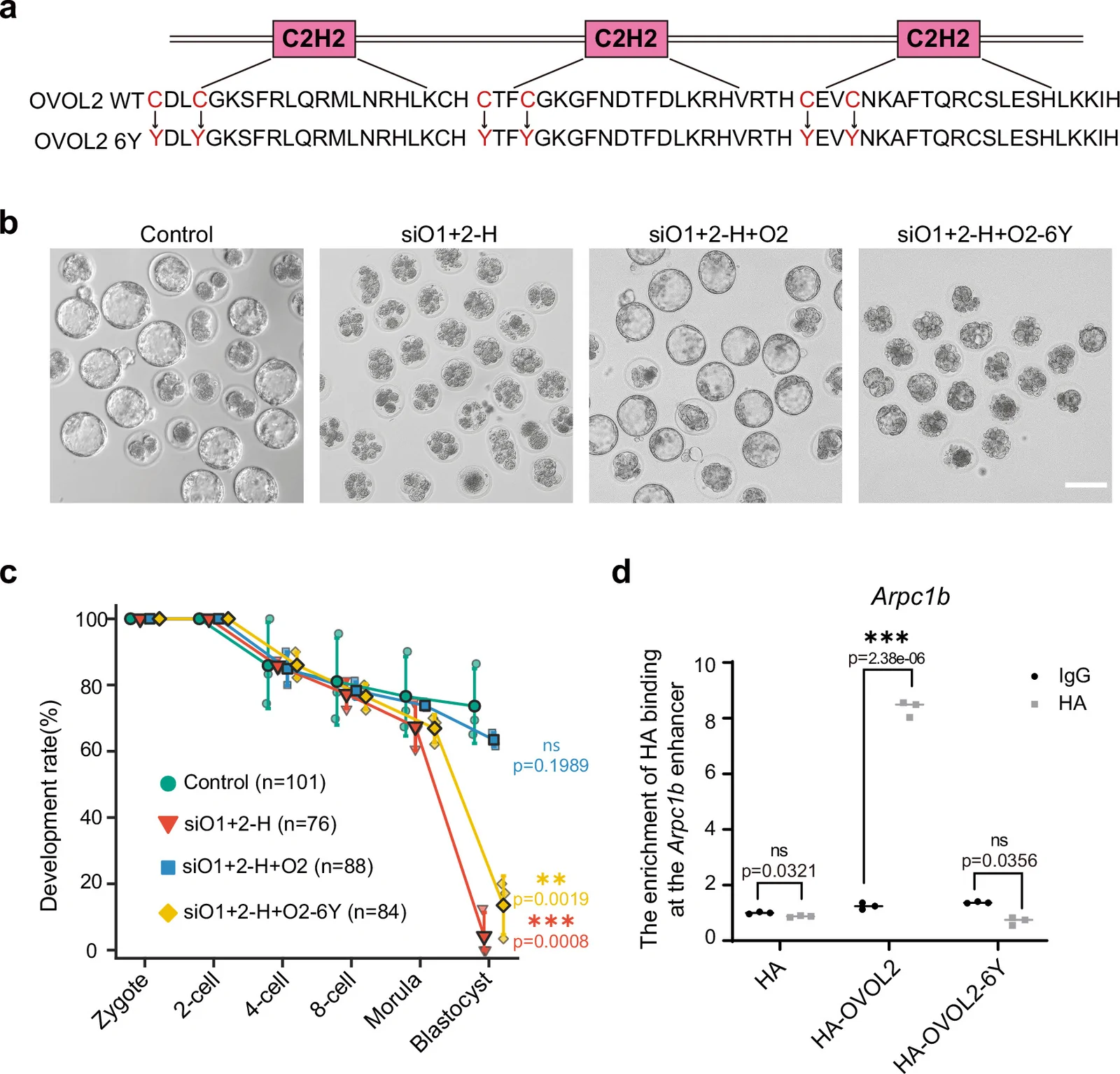
OVOL1/2 regulate ARPC1B transcription via zinc-finger domains. **a** Amino acid sequence of the OVOL2-6Y zinc-finger mutant. **b** Representative DIC images of E3.5 embryos following siRNA or siRNA+mRNA microinjection. Scale bar, 100 μm. **c** Developmental outcomes corresponding to (**b**). The number of embryos analyzed is indicated (n). Data are presented as mean ± SD. n.s. not significant, **P < 0.01, ***P < 0.001, by two-tailed Student’s *t*-test. **d** CUT&Tag-qPCR analysis of *Arpc1b* occupancy in 8-cell embryos expressing HA-tag, HA-OVOL2, or HA-OVOL2-6Y. IgG was used as a negative control. Data are presented as mean ± SD. n.s. not significant, ***P < 0.001, by two-tailed Student’s *t*-test.

Previous studies have shown that OVOL1/2 are zinc-finger transcription factors that bind DNA through their zinc-finger domains^36,37^. To determine whether this DNA-binding capacity is required for preimplantation development, we generated a mutant OVOL2 construct (OVOL2-6Y), in which cysteine residues within the zinc-finger motifs were substituted with tyrosine residues (Fig. 6a). Supplementation of siO1 + 2-H 4-cell embryos with OVOL2-WT or OVOL2-6Y mRNA revealed that OVOL2-WT, but not OVOL2-6Y, rescued the blastocyst formation defect caused by OVOL1/2 depletion (Fig. 6b, c). Furthermore, OVOL2-WT restored ARPC1B expression in siO1 + 2-H embryos, whereas OVOL2-6Y failed to do so (Supplementary Fig. 4b, c). To assess direct binding of OVOL1/2 to the *Arpc1b* locus, we performed CUT&Tag in embryos overexpressing HA-Tag, HA-OVOL2, or HA-OVOL2-6Y mRNA (Supplementary Fig. 4d). CUT&Tag–qPCR demonstrated robust enrichment of HA-OVOL2 at the *Arpc1b* locus relative to HA-Tag controls. This enrichment was abolished in embryos expressing the zinc-finger mutant HA-OVOL2-6Y and was absent in IgG controls (Fig. 6d). Together, these findings indicate that OVOL1/2 directly regulate *Arpc1b* transcription through their zinc-finger DNA-binding domains.

### OVOL1/2 are required for human blastocyst development

**Fig. 7:**
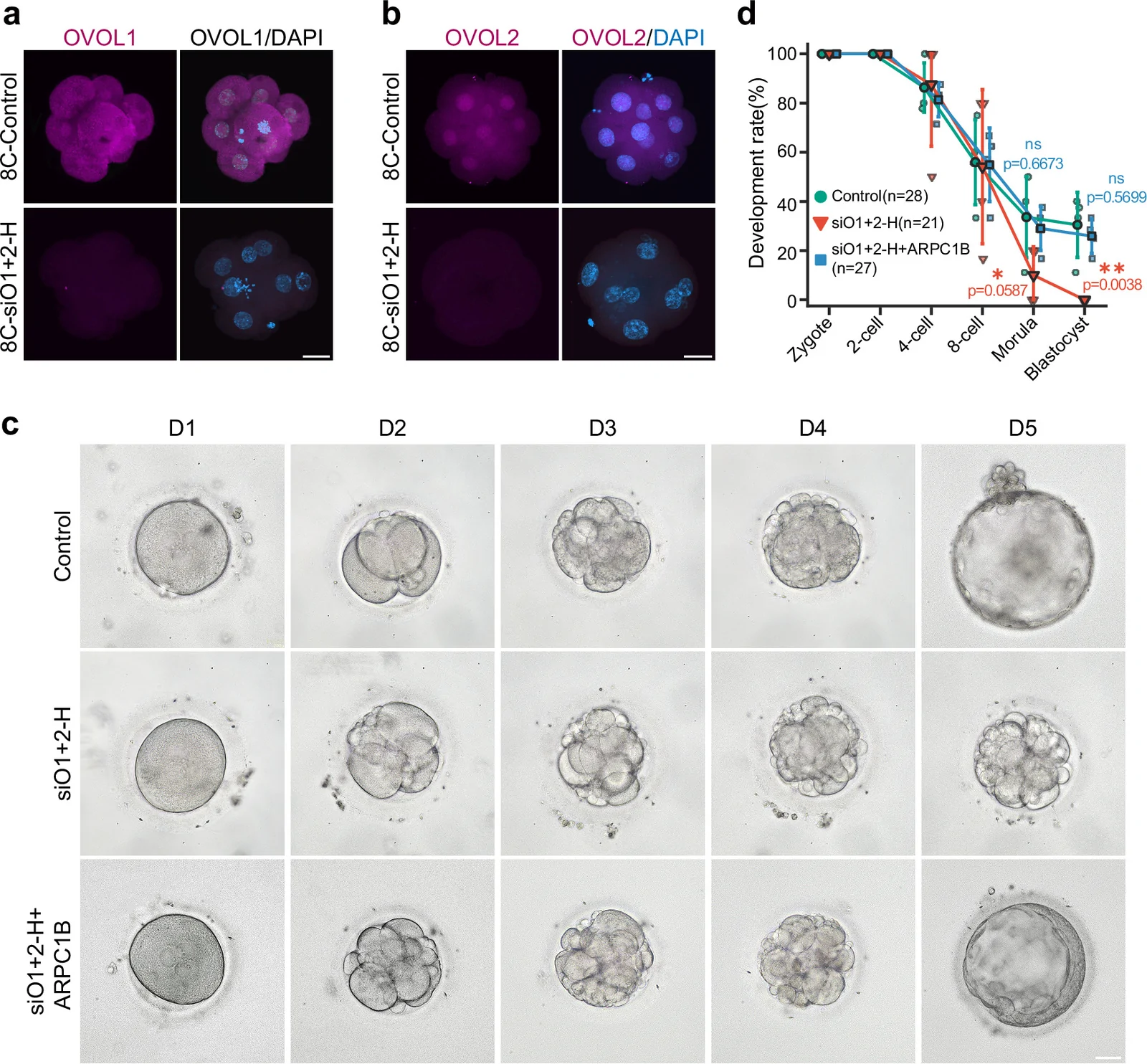
OVOL1/2 are required for human blastocyst formation. **a**,**b** Representative confocal images showing OVOL1 (**a**) and OVOL2 (**b**) expression in siO1 + 2 8-cell human embryos. DNA was counterstained with DAPI (blue). Scale bar, 25 μm. **c** Representative DIC images of human embryos at 0, 24, 48, 72, and 96 h post-injection. Scale bar, 100 μm. **d** Developmental rates corresponding to (**c**). The number of embryos analyzed is indicated (n). Data are presented as mean ± SD. n.s. not significant, *P < 0.05, **P < 0.01, by two-tailed Student’s *t*-test.

To investigate the role of OVOL1/2 in human preimplantation development, we microinjected tripronuclear (3PN) zygotes with siO1 + 2-H siRNAs, either alone or in combination with *ARPC1B* mRNA. OVOL1 and OVOL2 transcript levels were significantly reduced in the siO1 + 2-H embryos, confirming effective knockdown (Fig. 7a, b). Although no significant differences in developmental progression were observed up to the 8-cell stage, the rates of morula and blastocyst formation were markedly decreased in siO1 + 2-H embryos, with most embryos arresting at the morula stage. Notably, supplementation with *ARPC1B* mRNA partially rescued this phenotype, increasing the blastocyst formation rate to 25.9%, compared with 30.5% in the control group (Fig. 7c, d). Collectively, these findings demonstrate that OVOL1/2 are essential for human blastocyst development and that *ARPC1B* supplementation partially alleviates the developmental defects induced by OVOL1/2 depletion, consistent with observations in mouse embryos.

## Discussion

Disruption of ZGA genes is known to compromise progression from the morula to the blastocyst stage. For example, *Pabpn1* knockout in mice results in morula arrest due to defective maternal mRNA clearance^38^. NR5A2 is essential for morula development by promoting cell proliferation and maintaining genome stability^39^. Additional ZGA genes, including *TFAP2C*^40^, *TOE1*^41^, and *NOP2*^42^, have also been implicated in blastocyst formation. Here, we identify *OVOL1/2* as previously unrecognized, critical ZGA genes in early mouse and human embryos. Their suboptimal activation leads to failure of the morula-to-blastocyst transition. Mechanistically, OVOL1/2 regulate embryonic polarization and TE differentiation through ARPC1B. Collectively, these findings suggest that a cohort of ZGA genes, each executing distinct biological functions, cooperatively orchestrates blastocyst formation during preimplantation development.

In our study, individual knockdown of *Ovol1* or *Ovol2* exerted minimal effects on preimplantation development, whereas combined knockdown resulted in complete arrest at the morula-to-blastocyst transition, indicating functional redundancy, which is an established feature of early embryogenesis. For instance, loss of either *Hdac1* or *Hdac2* alone yields negligible phenotypes, whereas dual depletion leads to morula-stage lethality, accompanied by impaired lineage specification, increased apoptosis, and global DNA hypomethylation^43^. Similarly, simultaneous knockdown of *TPRX1* and *TPRX2* disrupts the activation of 15–21% of major ZGA genes, compared with only ~2% under individual knockdown conditions, underscoring their cooperative regulatory functions^8^. Such redundancy likely represents an evolutionarily conserved fail-safe mechanism that buffers against deleterious mutations^44^.

In OVOL1/2-deficient embryos, F-actin levels and the expression of genes required for polarization and compaction were markedly reduced at the morula stage, whereas the 8-cell stage was largely unaffected. This observation suggests that maternally derived factors may not yet be fully degraded at the 8-cell stage and may partially compensate for insufficient zygotic genome activation (ZGA). ARPC1B is a component of the ARP2/3 complex and is expressed from both maternal and embryonic sources. Inhibition of maternally derived ARP2/3 activity at the zygote stage induces cleavage-stage arrest, whereas insufficient activation of the embryonic ARP2/3 complex at the 8-cell stage impairs polarization, trophectoderm specification, and blastocyst formation^45^. These findings support a model in which maternal ARPC1B may initiate polarization, whereas embryonic ARPC1B is required for the maintenance of polarity. Given that OVOL1/2 predominantly regulate embryonic ARPC1B expression, OVOL1/2 knockdown leads to reduced embryonic ARPC1B transcription, thereby disrupting polarity maintenance rather than its initiation.

TFAP2C and TEAD4 are well-established regulators of embryonic polarization that promote apical domain assembly. However, premature overexpression of TFAP2C and TEAD4 induces polarization in only ~30% of 4-cell embryos^46^, suggesting that additional cooperating factors are required. Our data indicate that OVOL1/2 contribute to the establishment of embryonic polarity; however, whether they function through direct interactions with canonical polarity regulators or via the formation of functional molecular complexes remains to be determined. In addition, OVOL2 has been identified as a key repressor of aerobic glycolysis and a regulator of cancer metabolic reprogramming^47^. Metabolic cues, including desaturase-mediated modulation of membrane fluidity and cytoskeletal organization, have also been shown to promote polarization and blastocyst formation^48^. The precise molecular mechanisms by which OVOL1/2 regulate the establishment of early embryonic polarity remain to be fully elucidated.

## Methods

### Ethical statement

This study was conducted in accordance with all relevant ethical guidelines and regulatory requirements. The research protocol was reviewed and approved by the Academic and Ethics Committees of the Reproductive and Genetic Hospital of CITIC-XIANGYA (LL-SC-2023-023). All immature oocytes and 3PN zygotes were voluntarily donated by couples undergoing IVF treatment, with written informed consent obtained in accordance with standard clinical procedures^49^. All donors confirmed their voluntary participation without financial compensation. Individuals donating immature oocytes and 3PN zygotes were informed of the potential risks to their IVF treatment outcomes prior to consent.

### Collection and culture of human oocytes and embryos, and time-lapse imaging

Immature oocytes, identified by the absence of both a polar body and a visible germinal vesicle, were collected and cultured at 37.5 °C in a tri-gas incubator containing 6% CO₂, 5% O₂, and 89% N₂. Culture medium (Vitrolife) was supplemented with 0.075 IU/mL FSH, 0.5 IU/mL hCG, 1 mg/mL estradiol, and 0.5% human serum albumin. Only oocytes that extruded the first polar body within 4–8 h were selected for further use. Embryos were generated by intracytoplasmic sperm injection (ICSI) and cultured in pre-equilibrated EmbryoSlides (Vitrolife) containing G-1 Plus medium. Time-lapse imaging was performed using an EmbryoScope system, with images acquired at 10-min intervals. On day 3, the culture medium was exchanged and embryos were transferred to G-2 Plus medium for continued incubation. Blastocyst morphology was evaluated on day 5 (116 ± 2 h post-insemination) according to Gardner’s grading criteria^23^.

### Preparation and culture of mouse embryos

Wild-type C57BL/6 mice (Shanghai SLAC Laboratory Animal Co., Ltd.) were maintained under specific pathogen-free conditions at 20 °C–22 °C with a 12 h light/12 h dark cycle, 50–70% humidity, and ad libitum access to food and water. For superovulation, 4-week-old female mice received 5 IU pregnant mare serum gonadotropin (PMSG), followed 44 h later by 5 IU human chorionic gonadotropin (hCG) and mating with proven males. Mating was confirmed by the presence of a vaginal plug the next morning. Zygotes were collected from the oviducts and initially maintained in M2 medium (Merck) at 37 °C before transfer to KSOM medium (Sigma) under mineral oil. Embryos were harvested at 36, 48, 60, 72, and 84 h post-hCG, corresponding to the 2-cell, 4-cell, 8-cell, morula, and blastocyst stages, respectively.

### Embryo microinjection

Microinjection was performed using a micromanipulator mounted on an inverted microscope (Nikon Eclipse TE200). At the zygote stage, approximately 10 pL of synthetic mRNA (750 μg/mL) or siRNA (20 μM) was delivered into the cytoplasm. At the 2-cell stage, approximately 5 pL of synthetic mRNA (400 μg/mL) and siRNA (24 μM) was microinjected into the cytoplasm. The siRNA sequences are provided in Supplementary Table 2.

### Immunofluorescence staining

Embryos were treated with acidic Tyrode’s solution (Sigma) to remove the zona pellucida, then fixed in 4% paraformaldehyde (Sigma) for 30 min. After washing in PBS containing 0.1% BSA, embryos were permeabilized with 0.5% Triton X-100 (Sigma) for 30 min and blocked overnight at 4 °C in 5% normal donkey serum (Jackson ImmunoResearch) and 2% BSA. Primary antibody incubation was performed overnight at 4 °C, followed by washes and incubation with secondary antibodies and DAPI. Imaging was conducted on a Zeiss Celldiscoverer 7 with LSM 900. For quantification of F-actin and E-CAD fluorescence intensities, 8-cell embryos and morulae were segmented into individual blastomeres using the “Membrane-based Segmenter” module in Arivis. EGFP and Alexa Fluor 594 fluorescence signals were subsequently quantified for each blastomere. Based on EGFP fluorescence intensity, blastomeres were classified as EGFP-positive or EGFP-negative. Alexa Fluor 594 fluorescence intensities were then measured in both groups, and the fluorescence intensity ratio between EGFP-positive and EGFP-negative blastomeres was calculated. For pEzrin analysis, Alexa Fluor 594 fluorescence intensities in EGFP-positive and EGFP-negative blastomeres were manually quantified using ImageJ, followed by calculation of the fluorescence intensity ratio between the two groups. Antibodies are listed in Supplementary Table 3.

### Outgrowth assay

Blastocysts were individually cultured in DMEM (Gibco) supplemented with 10% FBS (Gibco) in Matrigel-coated 48-well plates (Corning) at 37 °C under 5% CO₂. Outgrowth morphology was assessed according to established criteria^50^.

### Single-embryo/-blastomere RNA-seq

On Day 3 (67–73 h post-ICSI), blastomeres were biopsied for single-cell RNA sequencing (scRNA-seq). Embryos were incubated in Ca²⁺/Mg²⁺-free medium for 2–5 min prior to zona pellucida opening using a laser system (HARIOMED), followed by isolation of individual blastomeres. Mouse embryos were processed in parallel by zona pellucida removal and dissociation into single cells. cDNA libraries were generated using the Smart-seq2 protocol^51^, and sequencing was performed on the BGISEQ-500 platform.

### Pre-processing and filtering of scRNA-seq data

Raw reads were quality-filtered using fastp (v0.20.0), aligned to GRCh38 (human) or GRCm38 (mouse) genomes using STAR (v2.7.8a), and quantified by StringTie (v2.0.4) using TPM (transcripts per million) as the metric for gene expression levels. Differential expression analysis was conducted using DESeq2, with significant genes defined as q < 0.05 and |log₂FC | ≥ 1. GO enrichment analysis was performed utilizing Metascape with its standard parameters (http://metascape.org/).

### Single-embryo RT-PCR

cDNA libraries were generated from two pooled embryos using Smart-seq2^51^. Quantitative RT-PCR was performed using SYBR Green Master Mix (Applied Biosystems), with *β-actin* serving as an internal reference gene. Relative expression levels were calculated using the 2⁻^ΔΔCT^ method. Primer sequences are listed in Supplementary Table 4.

### Western blotting

SDS-PAGE and immunoblotting were carried out according to standard protocols. Proteins were separated, transferred to membranes, blocked in 5% milk-TBST, and incubated with primary antibodies overnight at 4 °C. After secondary antibody incubation, signals were detected using ECL chemiluminescence. Antibody information is provided in Supplementary Table 3.

### Embryo electroporation

Electroporation was performed using custom platinum electrodes (BEX), as previously described^52^. A ~ 20-µm opening in the zona pellucida was created with a laser, and embryos were incubated in M2 medium containing RNA. Following electroporation, embryos were washed and cultured in KSOM medium at 37 °C under 5% CO₂.

### CUT&Tag-qPCR assay

CUT&Tag was carried out using a Hyperactive Universal CUT&Tag Kit (Vazyme Biotech). Mouse 8-cell embryos were incubated with concanavalin A-coated magnetic beads, followed by sequential incubation with primary and secondary antibodies. Transposase pA/G-Tnp was applied to cleave and fragment target DNA, which was subsequently purified and analyzed by qPCR. Antibodies and primer sequences are listed in Supplementary Tables 3 and 4.

### Statistical and reproducibility

Statistical analyses were performed using GraphPad Prism (version 10.1.2). All statistical tests were two-sided unless otherwise indicated. Data are presented as mean values ± SD. For comparisons between two groups, statistical significance was assessed using an unpaired two-tailed Student’s t-test. For comparisons among multiple groups, one-way or two-way analysis of variance (ANOVA) followed by appropriate post hoc multiple-comparison tests was performed. Significance was indicated as *P* < 0.05 (*), *P* < 0.01 (**), and *P* < 0.001 (***). A P-value < 0.05 was considered statistically significant unless otherwise specified. Sample sizes were determined based on previous studies and preliminary experiments. No statistical methods were used to predetermine sample sizes. Data points represent independent biological replicates unless otherwise stated. Biological replicates refer to independent experiments performed using independently generated samples. Technical replicates were not considered independent biological replicates. The specific statistical tests used for each experiment and exact P values are provided in the corresponding figure legends. All experiments were independently repeated at least three times unless otherwise stated. Representative images shown in the figures were selected from experiments that were independently replicated and showed consistent results. Experiments without statistical analysis, including representative microscopy images and qualitative assessments, were performed using multiple independent biological replicates to ensure reproducibility. All source data underlying the graphs presented in the main figures are provided in Supplementary Data.

## Supplementary information

Supplementary Information

## Data Availability

The raw sequence data reported in this paper have been deposited in the Genome Sequence Archive (Genomics, Proteomics & Bioinformatics 2021) of the National Genomics Data Center (Nucleic Acids Res 2022), China National Center for Bioinformation / Beijing Institute of Genomics, Chinese Academy of Sciences. Specifically, GSA-Human: HRA012432 serves as the source data for Fig. 1, while GSA-Mouse: CRA028031 serves as the source data for Fig. 3a-c, Fig. 4a-d, and Supplementary Fig. 3a-c. These data are publicly accessible at https://ngdc.cncb.ac.cn/gsa-human and https://ngdc.cncb.ac.cn/gsa. Public datasets from other studies are available in the Gene Expression Omnibus (GEO) repository under the following accession numbers: the scRNA-seq data of human and mouse pre-implantation embryos are from GSE36552, which serves as the source data for Supplementary Fig. 1b, and GSE106612, which serves as the source data for Fig. 5b and Supplementary Fig. 1a.
