# Supplementary material for: OVOL1/2 regulate trophectoderm differentiation during the morula-to-blastocyst transition
Source: Commun Biol. 2026 Oct 29;9:1386. doi: 10.1038/s42003-026-11168-x (PMC13720067; doi:10.1038/s42003-026-11168-x)
Supplement: Supplementary file 1 [file 42003_2026_11168_MOESM1_ESM.pdf]

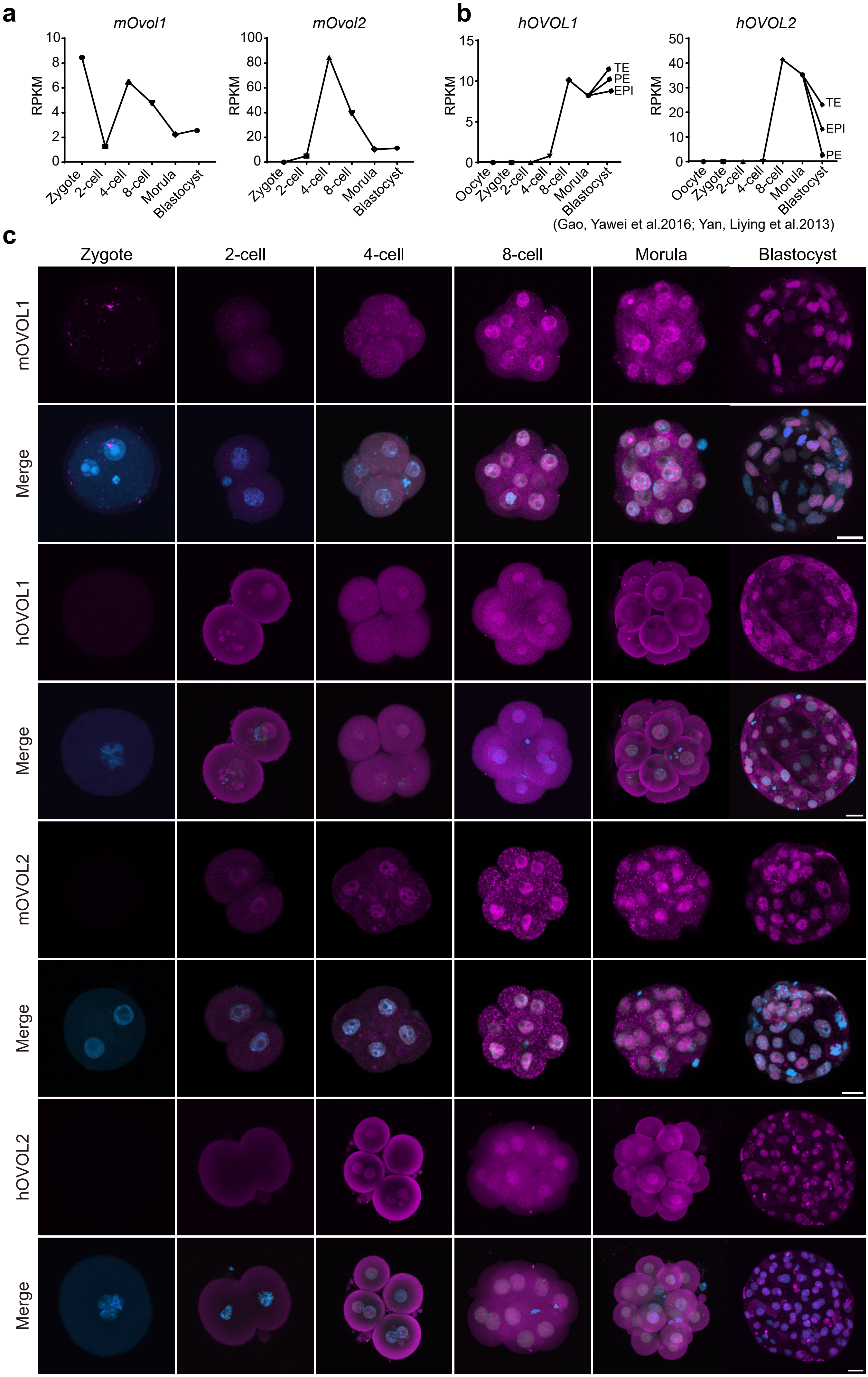

a
mOvol1
mOvol2
RPKM
Zygote
2-cell
4-cell
8-cell
Morula
Blastocyst
b
hOVOL1
hOVOL2
Oocyte
TE
PE
EPI
(Gao, Yawei et al.2016; Yan, Liying et al.2013)
c
Zygote
2-cell
4-cell
8-cell
Morula
Blastocyst
mOVOL1
Merge
hOVOL1
mOVOL2
hOVOL2

Figure S1. OVOL1/2 expression in mouse and human preimplantation embryos.

a, **b** RNA-seq analysis of *Ovol1* and *Ovol2* mRNA levels in mouse (**a**) and human (**b**) preimplantation embryos. TE, trophectoderm; PE, primitive endoderm; EPI, epiblast. RPKM values were derived from previously published datasets[21, 22]. **c** Representative confocal images showing OVOL1 and OVOL2 (magenta) immunofluorescence in mouse and human embryos from the zygote to the blastocyst stage. Human embryos were cultured from 3PN zygotes. DNA was counterstained with DAPI (blue). Scale bar, 25 μm.

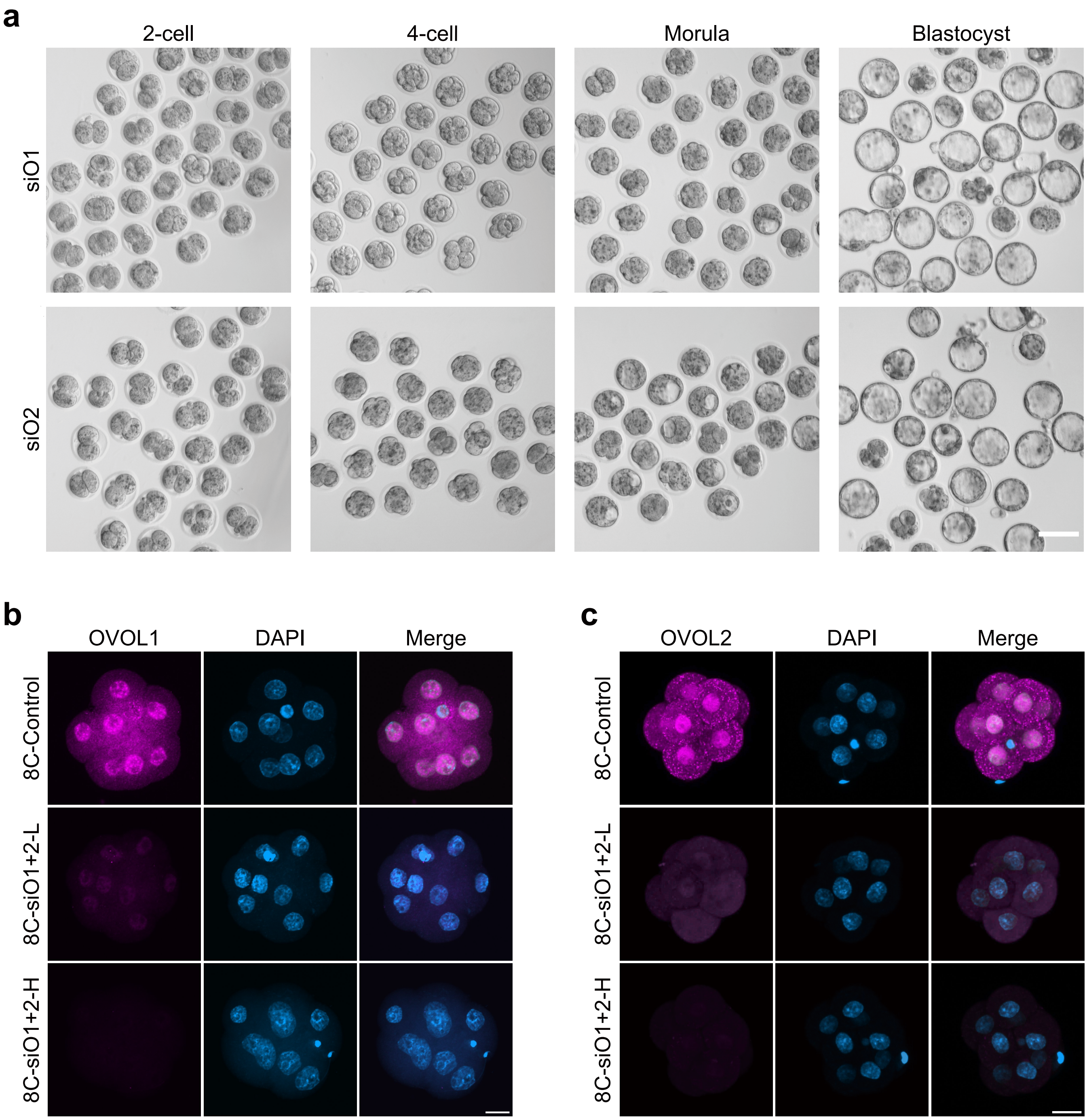
a
2-cell
4-cell
Morula
Blastocyst
siO1
siO2
b
OVOL1
DAPI
Merge
8C-Control
8C-siO1+2-L
8C-siO1+2-H
c
OVOL2
DAPI
Merge
8C-Control
8C-siO1+2-L
8C-siO1+2-H

Figure S2. Individual knockdown of *Ovol1* or *Ovol2* does not impair blastocyst formation.

a Representative DIC images of embryos at the 2-cell, 4-cell, morula, and blastocyst stages following the indicated siRNA treatments. Scale bar, 100 μm. **b**, **c** Representative confocal images showing OVOL1 (**b**) and OVOL2 (**c**) immunofluorescence (magenta) in siO1+2 8-cell embryos. DNA was counterstained with DAPI (blue). Scale bar, 25 μm.

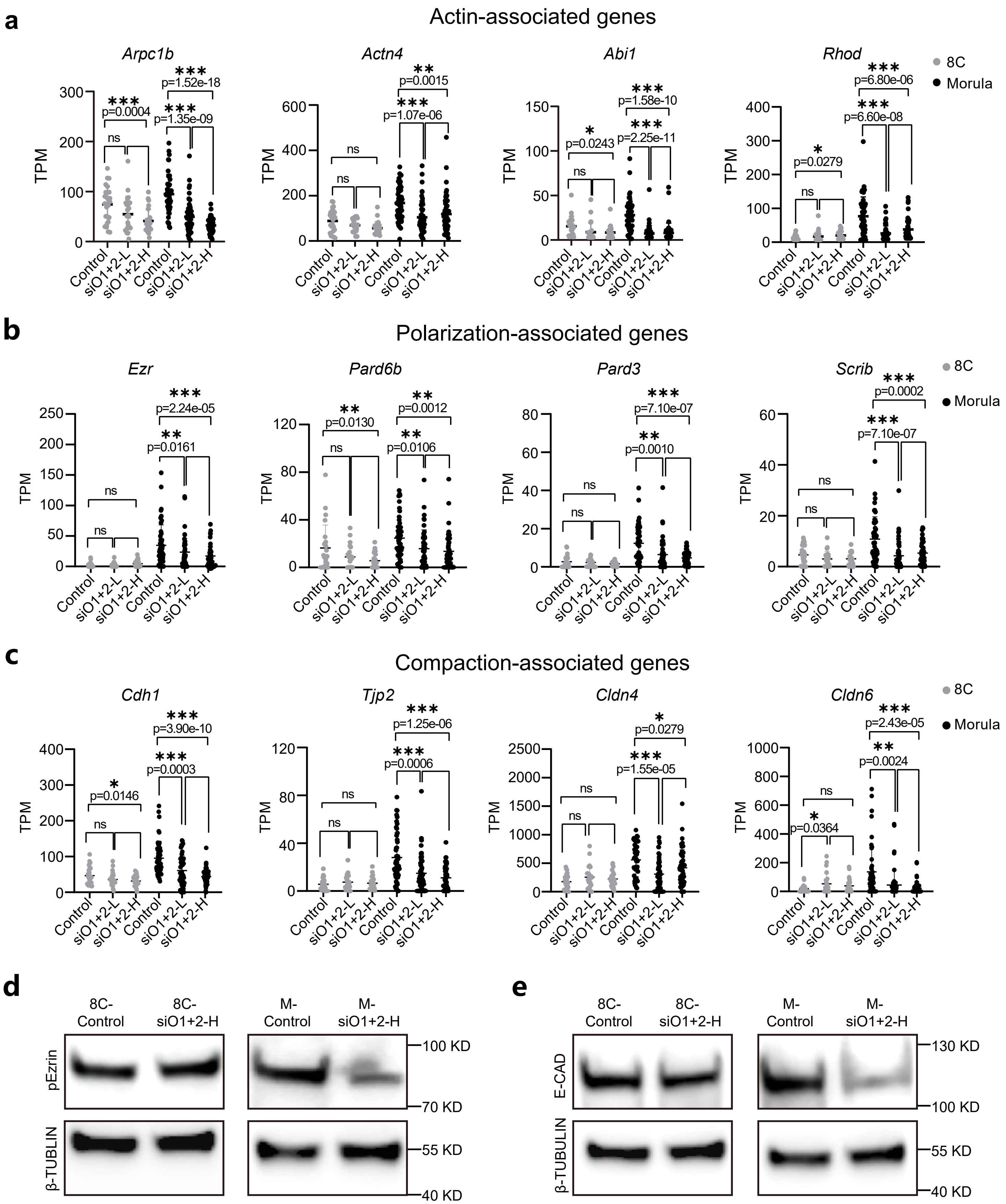
a
Actin-associated genes
Arpc1b
Actn4
Abi1
Rhod
8C
Morula
TPM
Control
siO1+2-L
siO1+2-H
ns
***
p=0.0004
p=1.52e-18
p=1.35e-09
**
p=0.0015
p=1.07e-06
*
p=0.0243
p=1.58e-10
p=2.25e-11
p=0.0279
p=6.80e-06
p=6.60e-08
b
Polarization-associated genes
Ezr
Pard6b
Pard3
Scrib
p=2.24e-05
p=0.0161
p=0.0130
p=0.0012
p=0.0106
p=7.10e-07
p=0.0010
p=0.0002
p=7.10e-07
c
Compaction-associated genes
Cdh1
Tjp2
Cldn4
Cldn6
p=0.0146
p=3.90e-10
p=0.0003
p=1.25e-06
p=0.0006
p=0.0279
p=1.55e-05
p=0.0364
p=2.43e-05
p=0.0024
d
8C-Control
8C-siO1+2-H
M-Control
M-siO1+2-H
pEzrin
β-TUBLIN
100 KD
70 KD
55 KD
40 KD
e
E-CAD
130 KD
100 KD

Figure S3. OVOL1/2 knockdown impairs embryonic polarization and compaction.

a, **b**, **c** Scatterplots showing the expression levels of actin-associated (**a**), polarization-associated (**b**) and compaction-associated (**c**) genes in control and siO1+2 embryos. Data are presented as mean ± S.D. TPM, transcripts per million. **d**, **e** Western blot analysis of pEzrin (**d**) and E-CAD (**e**) protein levels in control and siO1+2-H 8-cell and morula-stage embryos. β-Tubulin was used as a loading control. Total protein lysates from 100 embryos were loaded per lane. Molecular weights (kDa) are indicated on the right.

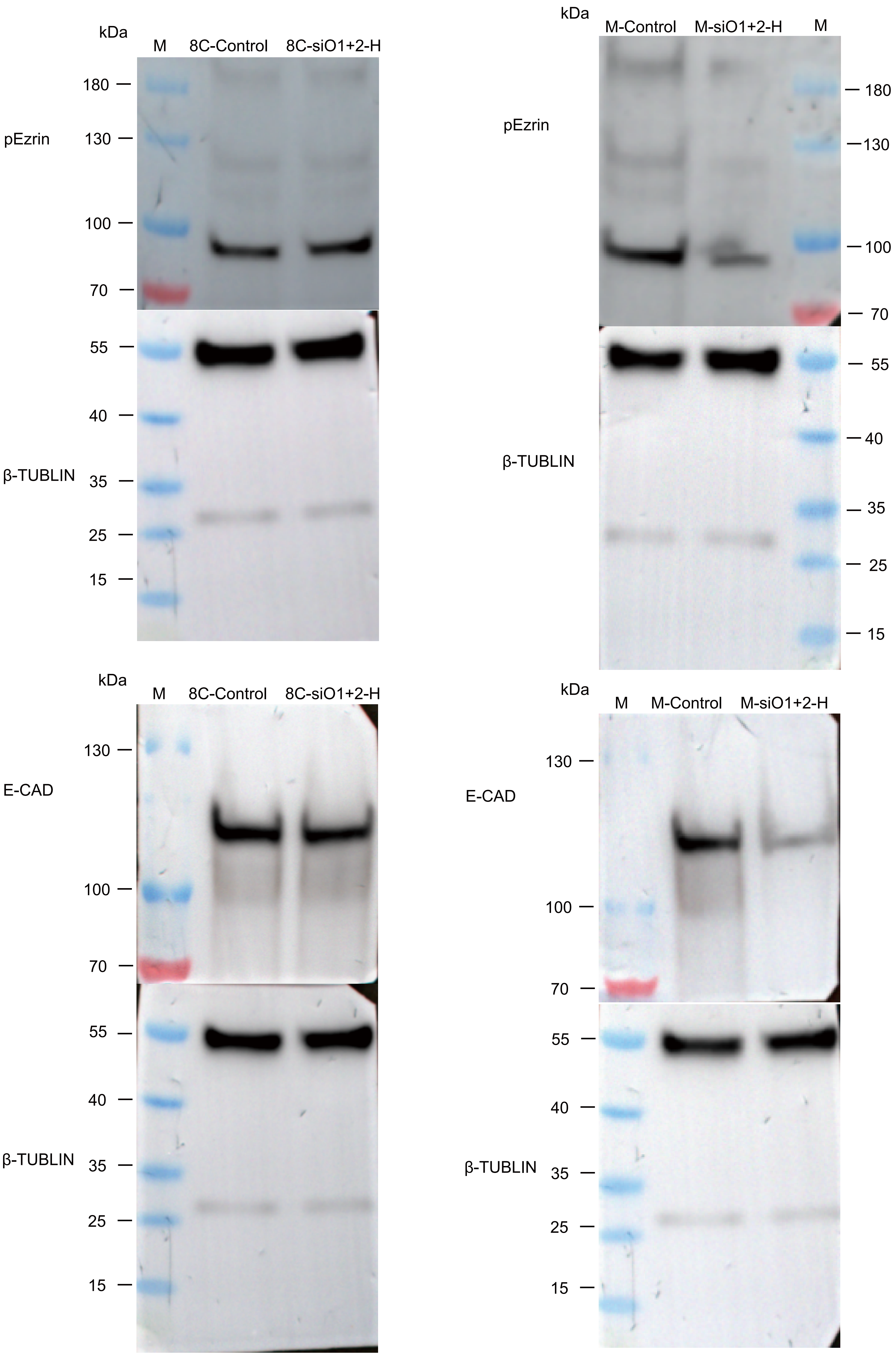

kDa
M
8C-Control
8C-siO1+2-H
pEzrin
180
130
100
70
55
40
β-TUBLIN
35
25
15
kDa
M-Control
M-siO1+2-H
M
pEzrin
180
130
100
70
55
40
β-TUBLIN
35
25
15
kDa
M
8C-Control
8C-siO1+2-H
E-CAD
130
100
70
55
40
β-TUBLIN
35
25
15
kDa
M
M-Control
M-siO1+2-H
E-CAD
130
100
70
55
40
β-TUBLIN
35
25
15

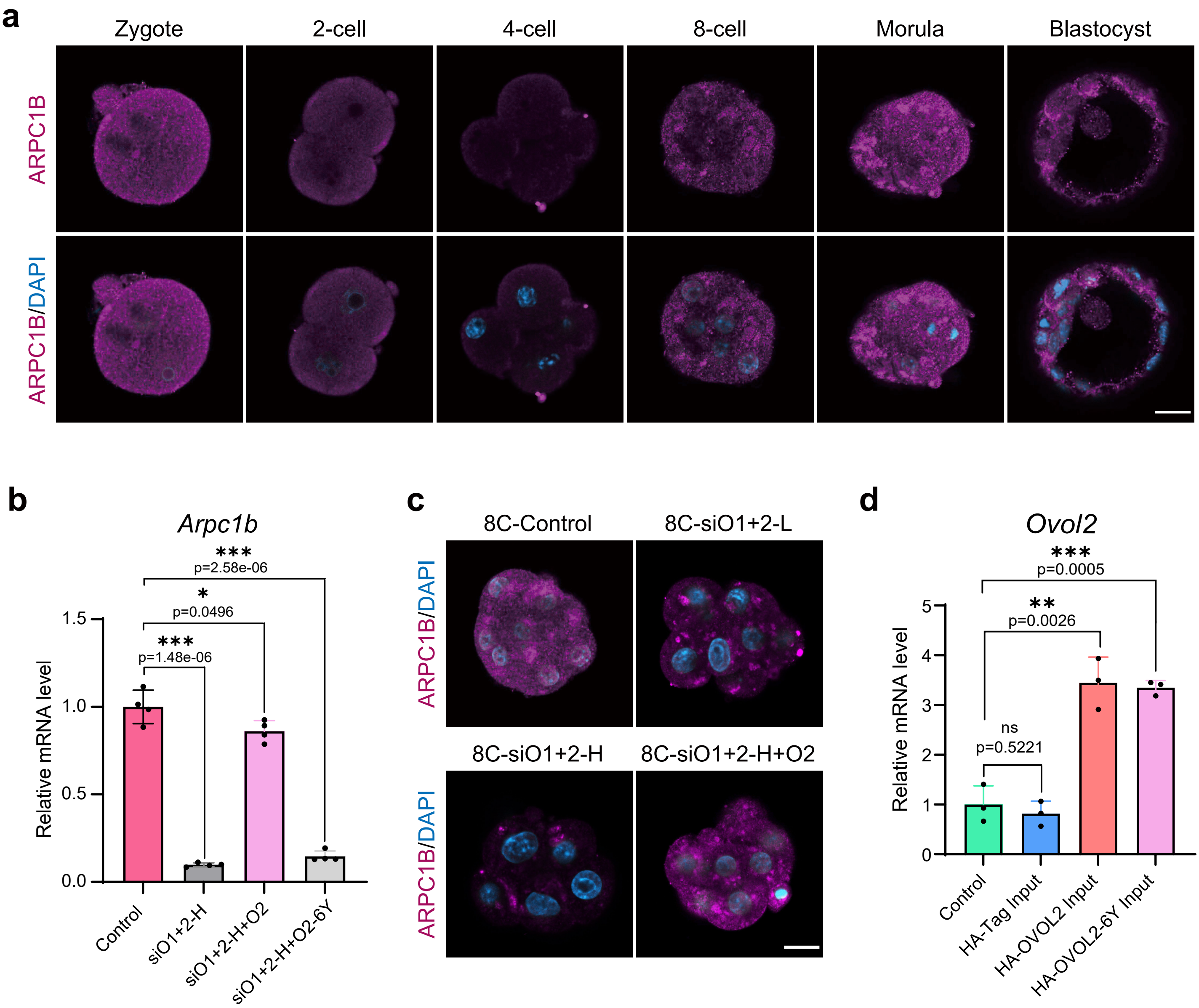
a
Zygote
2-cell
4-cell
8-cell
Morula
Blastocyst
ARPC1B
ARPC1B/DAPI
b
Arpc1b
***
p=2.58e-06
*
p=0.0496
***
p=1.48e-06
Relative mRNA level
1.5
1.0
0.5
0.0
Control
siO1+2-H
siO1+2-H+O2
siO1+2-H+O2-6Y
c
8C-Control
8C-siO1+2-L
8C-siO1+2-H
8C-siO1+2-H+O2
ARPC1B/DAPI
ARPC1B/DAPI
d
Ovol2
***
p=0.0005
**
p=0.0026
ns
p=0.5221
Relative mRNA level
5
4
3
2
1
0
Control
HA-Tag Input
HA-OVOL2 Input
HA-OVOL2-6Y Input

Figure S4. OVOL1/2 regulate ARPC1B expression via zinc-finger domains.

a Representative confocal images showing ARPC1B (magenta) expression in preimplantation embryos. DNA was counterstained with DAPI (blue). Scale bar, 25 μm. **b** RT-PCR analysis of *Arpc1b* mRNA levels at the 2-cell stage following siRNA or siRNA+mRNA injection. *β-actin* was used as an internal control (n = 2 embryos per sample; three biological replicates). Data are presented as mean ± S.D. and normalized to control (set to 1.0). **c** Representative confoca images showing ARPC1B (magenta) expression in 8-cell embryos following siRNA or siRNA+mRNA injection. DNA was counterstained with DAPI (blue). Scale bar, 25 μm. **d** RT-PCR analysis of *Ovol2* mRNA levels at the 8-cell stage following mRNA injection. *β-actin* was used as an internal control (two biological replicates). Data are presented as mean ± S.D. and normalized to control (set to 1.0).

Supplementary Table 1. Comparison of morphokinetic parameters between HQBs and BFEs

Morphokinetic parameters	HQBs (n=21)		BFEs (n=5)		***P***
	**Average**	**SD**	**Average**	**SD**	
t2	26.26	1.92	27.40	1.56	0.2312
t4	37.92	1.93	38.60	1.88	0.4839
ECC2 (t4–t2)	11.66	0.90	11.20	0.49	0.2851
t8	54.96	3.39	57.80	4.09	0.1176
ECC3 (t8–t4)	17.04	2.58	19.20	3.47	0.1273
tSC	84.61	8.16	98.72	5.95	0.0014
tM	88.85	7.32	101.78	7.38	0.0016
tM-t8	33.89	6.50	43.98	8.02	0.0063
tSB	101.31	7.35	115.73	6.89	0.0006
tSB–tM	12.35	4.73	16.07	9.77	0.2156

Supplementary Table 2. siRNA sense sequence information

Name	Species	Sequence
siControl	Mouse & Human	5’- UUCUCCGAACGUGUCACGUTT -3’
si*Ovol1*_1	Mouse	5’- CCTTCGAGACTCCAGCTAT -3’
si*Ovol1*_2	Mouse	5’- GCAGCAGAAGTATGCATAC -3’
si*Ovol1*_3	Mouse	5’- GCGCAAGACCTCCAAGAAA -3’
si*Ovol2*_1	Mouse	5’- GCACATGCGACAACTCTGT -3’
si*Ovol2*_2	Mouse	5’- GCAGCAGTATGCCTACAAG -3’
si*Ovol2*_3	Mouse	5’- CCCTTATGCAGAACAAGCT -3’
si*Arpc1b*_1	Mouse	5’- CAAGAACAGTGTCAGCCAA -3’
si*Arpc1b*_2	Mouse	5’- GTACACTGCTACAAAGGAA -3’
si*Arpc1b*_3	Mouse	5’- GGACCTGAAAATCAAATGA -3’
si*Ovol1*_1	Human	5’- CCTTCGACCTCAAGAGACA -3’
si*Ovol1*_2	Human	5’- GCACTACAGAACACTGTCA -3’
si*Ovol1*_3	Human	5’- GGGAAGGGCTTCAATGACA -3’
si*Ovol2*_1	Human	5’- CAGCAGCAGTATGCCTATA -3’
si*Ovol2*_2	Human	5’- ACCTGTACCTGCACGTGAA -3’
si*Ovol2*_3	Human	5’- GCACACCAGGAGAATACCA -3’

Supplementary Table 3. Antibody information

Antibody name	Manufacture (catalogue number)	Applications (working dilution)
anti-NANOG	Abcam (#ab109250)	IF (1:50)
anti-SOX17	R&D systems (#AF1924)	IF (1:40)
anti-OVOL1	YouKe (#4917)	IF (1:100)
anti-OVOL2	Invitrogen (#PA5-115700)	IF (1:200)
anti-Phalloidin (F-actin)	Invitrogen (#A22287)	IF (1:300)
anti-pEzrin (Thr567)	CST (#3726)	IF (1:200)
anti-E-CAD	CST (#3195)	IF (1:500)
anti-OCT4	Santa (#sc-5279)	IF (1:200)
anti-CDX2	Biogenex (#MU392A-UC)	IF (1:100)
anti-CDX2	R&D systems (#AF3665)	IF (1:200)
anti-HA tag	Invitrogen (#26183)	IP (1:100)<br>WB (1:1000)
anti-IgG	Sigma (#12-370)	IP (1:100)
anti-ARPC1B	Novus Biologicals (#NBP2-15262)	IF (1:200)
anti-FLAG tag	Invitrogen (#MA1-91878)	WB (1:1000)
anti-His tag	Proteintech (#66005)	WB (1:1000)
Alexa Fluor 488 donkey anti-mouse IgG	Thermo Fisher Scientific (#A_21202)	IF (1:500)
Alexa Fluor 488 donkey	Thermo Fisher Scientific	IF (1:500)

anti-rabbit IgG	(#A_21206)	
Alexa Fluor 594 donkey anti-rabbit IgG	Thermo Fisher Scientific (#A_A21207)	IF (1:500)
Alexa Fluor 647 donkey anti-goat IgG	Thermo Fisher Scientific (#A_A21447)	IF (1:500)
HRP-conjugated Goat anti-mouse IgG	Proteintech (#SA00001-1)	WB (1:1000)
HRP-conjugated Goat anti-rabbit IgG	Proteintech (#SA00001-2)	WB (1:1000)

Supplementary Table 4. Real-time PCR primers information

**Name**	**Species**	**Sequence**
Ovol1-FP	Mouse	AGCCTTCGAGACTCCAGCTA
Ovol1-RP	Mouse	AGGTCACCTTCATCTTGGTTCG
Ovol2-FP	Mouse	CCGGAGCCTGAAACTCCAG
Ovol2-RP	Mouse	CATGTGCCGGTGGTAAACT
β-actin-FP	Mouse	CCTCTATGCCAACACAGTGC
β-actin-RP	Mouse	CCTGCTTGCTGATCCACATC
Arpc1b-FP	Mouse	TCATTTGATTTTCAGGTCCTTCAA
Arpc1b-RP	Mouse	ATGGCCTACCACAGCTTCCTGGTG
